# *CYP2C*:*TG* Haplotype in Native Mexicans, Molecular Ancestry and Its Implications for *CYP2C19* Genotype–Phenotype Correlation

**DOI:** 10.3390/ph19010006

**Published:** 2025-12-19

**Authors:** Carla González de la Cruz, Nadine de Godoy Torso, Juan Antonio Villatoro-García, Carmen Mata-Martín, Fernanda Rodrigues-Soares, Carlos Galaviz-Hernández, Eva Peñas-Lledó, Martha Sosa-Macías, Adrián LLerena, RIBEF-IBEROFEN Consortium

**Affiliations:** 1 RIBEF/SIFF Red y Sociedad Iberoamericana de Farmacogenética y Farmacogenómica, 06080 Badajoz, Spain; carla.gonzalezd@externos.salud-juntaex.es (C.G.d.l.C.); nadinetorso@gmail.com (N.d.G.T.); juan.villatorogarcia@externos.salud-juntaex.es (J.A.V.-G.); mariadelcarmen.mata@salud-juntaex.es (C.M.-M.); fernanda.soares@uftm.edu.br (F.R.-S.); carlosgalavizhernandez55@gmail.com (C.G.-H.); elledo@unex.es (E.P.-L.); 2 University Institute for Bio-Sanitary Research of Extremadura (INUBE), 06080 Badajoz, Spain; 3 Facultad de Ciencias Médicas, Universidade Estadual de Campinas, Campinas 13083-887, Brazil; 4 Department of Pathology, Genetic and Evolution, Universidade Federal do Triângulo Mineiro, Uberaba 38025-180, Brazil; 5 Academia de Genómica, Instituto Politécnico Nacional, CIIDIR Unidad Durango, Durango 34220, Mexico; 6 Faculty of Medicine and Health Sciences, University of Extremadura, 06006 Badajoz, Spain

**Keywords:** *CYP2C*:*TG* haplotype, CYP2C19, molecular ancestry, native Mexican populations, pharmacogenetics

## Abstract

**Background:** Recent studies have associated the presence of the *CYP2C*:*TG* haplotype with increased metabolism of CYP2C19 substrates such as escitalopram and sertraline, suggesting a potential regulatory interaction between *CYP2C18* and *CYP2C19*. However, this association has not been demonstrated for other CYP2C19 substrates. **Objective:** This study aims to elucidate the role of the *CYP2C*:*TG* haplotype in modulating CYP2C19 activity using the omeprazole metabolic ratio (MR) within a cocktail drug approach, to characterize its distribution and prevalence among Native Mexican populations, and to evaluate its potential impact on CYP2C19 metabolic phenotypes. **Materials and Methods:** A total of 256 volunteers from various ethnic native groups from Mexico were genotyped for *CYP2C19* (*2, *3, *4, *5, *17) and the *CYP2C* haplotype (rs2860840 and rs11188059). The MR of omeprazole to 5-hydroxyomeprazole was analyzed to determine individual CYP2C19 metabolic phenotypes and assess metabolic capacity. **Results:** The *CYP2C*:*TG* haplotype was the most prevalent (42.77%), followed by *CYP2C*:*CG* (35.74%) and *CYP2C*:*TA* (21.48%). The *CYP2C*:*TG* haplotype was consistently associated with the CYP2C19*1 allele. Significant differences in logMR values were observed between individuals with and without the *TG* haplotype (*p* = 0.02). A trend toward increased metabolic activity associated with *CYP2C*:*TG* was observed across most CYP2C19 metabolizer groups, except for rapid metabolizers. No significant association was found between molecular ancestry and the presence or functionality of the haplotype. **Conclusions:** The *CYP2C*:*TG* haplotype appears to be associated with increased CYP2C19 activity, warranting further functional validation before clinical implementation.

## 1. Introduction

The *CYP2C* locus includes four cytochrome P450 genes: *CYP2C8*, *CYP2C9*, *CYP2C18*, and *CYP2C19* [1]. The CYP2C19 enzyme plays a key role in the metabolism of a wide range of clinically relevant drugs, including tricyclic antidepressants, selective serotonin reuptake inhibitors, antiplatelet agents, and proton pump inhibitors such as omeprazole.

Nucleotide variants within the *CYP2C19* gene are well-established determinants of interindividual variability in drug response [2]. These genetic variants result in distinct CYP2C19 metabolic phenotypes, categorizing individuals into different metabolizer groups [3], and have been incorporated into clinical pharmacogenetic guidelines such as those for clopidogrel, omeprazole or antidepressants to optimize therapy [4,5,6]. However, no clinical guidelines currently exist for drugs metabolized by CYP2C18, despite evidence of its involvement in the metabolism of acenocoumarol, clobazam, diclofenac, and warfarin, as reported by ClinPGx [7].

A novel haplotype, the *CYP2C*:*TG*, which consists of two non-coding variants in the *CYP2C18* gene, rs2860840 (NM_000772.3c.31C > T) and rs11188059 (NM_000772.2c.819 + 2182G > A) [8], has been associated with increased metabolic activity of CYP2C19 substrates, suggesting a potential regulatory interplay between *CYP2C18* and *CYP2C19* [8]. Initial findings linked the *CYP2C*:*TG* haplotype to ultrarapid metabolism of escitalopram and sertraline in European populations [8,9].

However, subsequent studies involving CYP2C19 substrates such as escitalopram [8] and clopidogrel [10], as well as investigations assessing in vivo drug exposure, CYP2C19 protein abundance and enzyme activity in human liver tissue [11], have failed to demonstrate any significant contribution of the *CYP2C*:*TG* haplotype to the metabolism of drugs primarily processed by CYP2C19. Thus, the mechanism underlying the increase in metabolic activity remains unclear.

On the other hand, the haplotype’s frequency varies widely across populations, suggesting ancestry-dependent effects and possible linkage with other variants [12]. While the haplotype may influence CYP2C19 metabolic phenotypes, particularly in populations such as Native Americans, where conventional gain-of-function alleles such as CYP2C19*17 are rare or absent, its clinical relevance requires further evaluation across diverse cohorts before it can be integrated into clinical pharmacogenetic practice [13].

Given the growing interest in the *CYP2C*:*TG* haplotype and its potential role in drug metabolism, this study aims to evaluate the role of this variant in modulating CYP2C19 activity, assessed through the omeprazole metabolic ratio (MR). The relationship between the *CYP2C*:*TG* haplotype and MR, as well as its prevalence across different genomic ancestry groups in Mexicans, will be studied.

## 2. Results

This section is divided by subheadings. It provides a concise and precise description of the experimental results, their interpretation, as well as the experimental conclusions that can be drawn.

### 2.1. CYP2C19 Genotype and CYP2C Haplotype

The genotype frequencies were consistent with Hardy–Weinberg equilibrium (*p* = 0.15). Among the possible haplotype combinations involving rs2860840C > T and rs11188059G > A, the *CYP2C*:*TG* haplotype was the most prevalent, with a frequency of 42.77%. The *CYP2C*:*CG* and *CYP2C*:*TA* haplotypes were observed at frequencies of 35.74% and 21.48%, respectively. Notably, the *CYP2C*:*CA* haplotype was absent in this cohort.

Table 1 presents the distribution of *CYP2C19* genotypes in conjunction with the three observed *CYP2C* haplotypes. The *CYP2C*:*TG* haplotype was predominantly associated with the CYP2C19*1 allele (42.58%) and was not detected in combination with the CYP2C19*17 allele. The CYP2C19*2 allele was most frequently linked to the *CYP2C*:*CG* haplotype, with only one exception, whereas the CYP2C19*17 allele was exclusively found in combination with *CYP2C*:*CG*.

### 2.2. Effect of CYP2C19 Genotypes and CYP2C Haplotype on logMR

**Figure 1:**
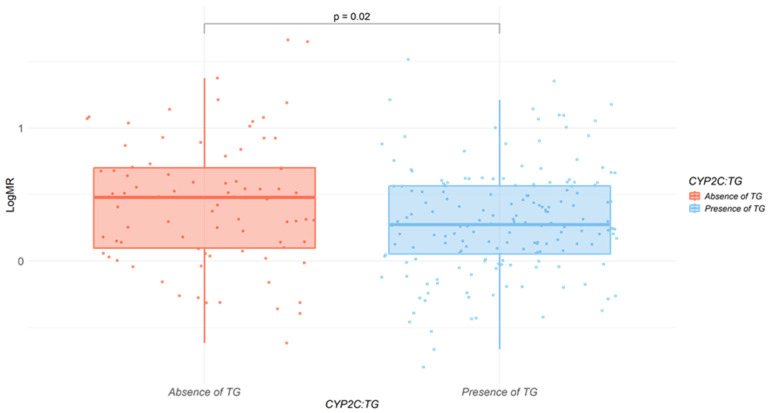
Relationship between the presence of *CYP2C*:*TG* and logMR. Boxplot illustrating logMR levels according to the presence (red) or absence (blue) of the *CYP2C*:*TG* haplotype. The diagram also displays the *p*-obtained from the Wilcoxon rank-sum test, indicating the statistical significance of the observed differences.

The presence of the *CYP2C*:*TG* haplotype was significantly associated with logMR of omeprazole/5-hydroxyomeprazole, as evidenced by a *p*-value of 0.02 (Figure 1).

In the process of inferring individual haplotypes and diplotypes derived from *CYP2C19* star alleles and *CYP2C* genetic variants, the diplotype predominantly identified was *1/*1, in association with the *CYP2C*:*TG* haplotype either in a homozygous *1+TG/*1+TG (17.9%) or a heterozygous state *1/*1+TG (37.7%), representing a 55.6% sum within the study population. Individuals carrying the *1/*2 genotype and the *CYP2C*:*TG* haplotype in homozygosity (0.4%) or heterozygosity (9.7%) accounted for 10.1% of the cohort, while 1.6% of individuals were identified as carriers of the *1+TG/*17 diplotype.

The distribution of the *CYP2C*:*TG* haplotype across age groups was examined to assess whether age influences its functional impact (Table 2). The *1+TG/*17 genotype was associated with the oldest age subgroup, which also demonstrated the highest logMR values.

In the multivariate analysis, the diplotype and age were the most relevant factors influencing MR.

**Figure 2:**
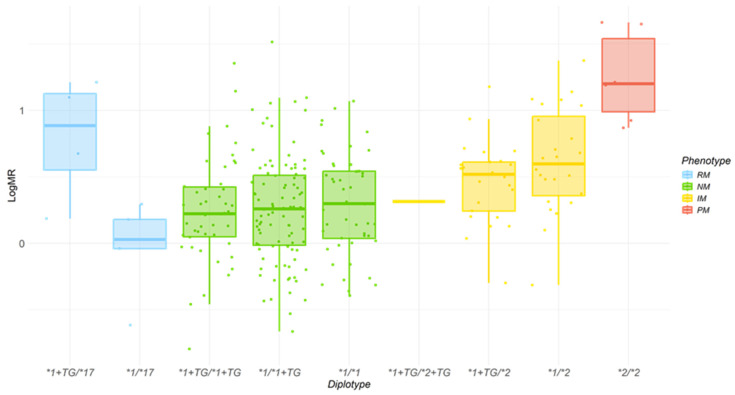
Association between logMR and the presence of the *CYP2C*:*TG* haplotype, stratified by *CYP2C19* diplotype distribution. Boxplot illustrating logMR levels across combinations of *CYP2C19* genotypes and the presence or absence of the *CYP2C*:*TG* haplotype. Box colors indicate predicted metabolic phenotypes: red for gPMs, yellow for gNMs, green for gIMs and blue for gRMs.

An association was observed between metabolizer phenotypes, defined by *CYP2C* diplotypes, and the resulting logMR (Figure 2). Individuals classified as gIMs, or gNMs exhibited enhanced metabolic capacity when carrying the *CYP2C*:*TG* haplotype, regardless of zygosity (homozygous or heterozygous). In contrast, gRMs did not display the increase in enzymatic activity, showing a MR comparable to that of gIMs individuals.

The analysis of the association between logMR and the combined presence of *CYP2C19* genotype and the *CYP2C*:*TG* haplotype revealed several trends. Individuals with the CYP2C19*1/*1 genotype carrying one copy of the *CYP2C*:*TG* haplotype exhibited a 14.7% reduction in mean logMR compared to non-carriers, although this difference was not statistically significant (*p* = 0.43). Similarly, carriers of the two copies of the *CYP2C*:*TG* haplotype showed a 17.4% decrease in mean logMR relative to the baseline group (*p* = 0.45). Among individuals with the CYP2C19*1/*2 genotype, the presence of the CYP2C19*1 allele in combination with the *CYP2C*:*TG* haplotype was associated with a 25.8% reduction in mean logMR (*p* = 0.16). In contrast, only four individuals carrying the CYP2C19*1/*17 genotype showed an opposite effect: the presence of the combination of CYPC19*1/*17 and the *CYP2C*:*TG* haplotype was associated with an increase in mean logMR compared to the baseline group (*p* = 0.032).

### 2.3. Relationship Between Ancestry and Metabolic Ratio

**Figure 3:**
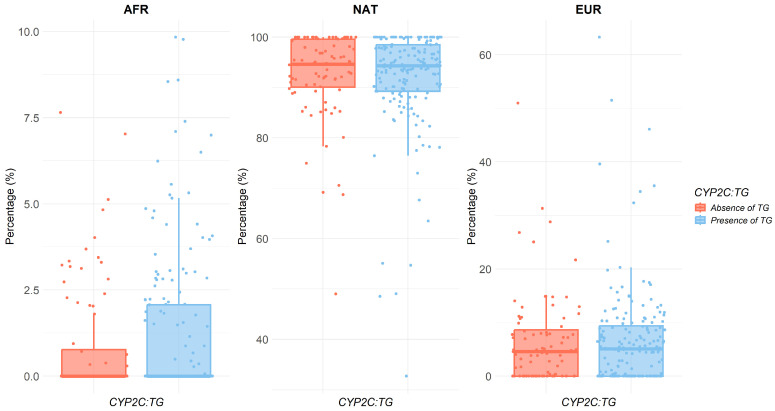
Association between the presence of the *CYP2C*:*TG* haplotype and genomic ancestry. Boxplots showing the proportion of ancestral components—African (AFR), European (EUR), and Native American (NAT)—stratified by the presence (red) or absence (blue) of the *CYP2C*:*TG* haplotype.

The analyzed Mexican Amerindian population exhibited a molecular ancestry composition of 92.12% Native American, 6.77% European, and 1.11% African. The high proportion of Native American ancestry was evaluated in relation to the presence of the *CYP2C*:*TG* haplotype. However, no statistically significant differences were observed across ancestry components (AFR *p* = 0.35, NAT *p* = 0.8, EUR *p* = 0.98), suggesting that molecular ancestry does not influence haplotype carriage (Figure 3, Table 3).

When comparing the mean ancestry proportions among individuals carrying the three possible *CYP2C* haplotypes, statistically significant differences were observed only for African ancestry, specifically between carriers of the *CYP2C*:*TA* haplotype and non-carriers.

## 3. Discussion

This study presents, for the first time, the relationship between the novel *CYP2C* haplotype and its potential contribution to predicting the CYP2C19 metabolic phenotype in a Native Mexican population, using a validated phenotyping cocktail approach [14,15]. The *CYP2C* haplotype, defined by two SNPs (rs2860840 and rs11188059), has been previously associated with significantly lower serum concentration of escitalopram [8] and sertraline [9]. These findings support the hypothesis that the *CYP2C*:*TG* haplotype is associated with ultrarapid CYP2C19 metabolism [9]. Nevertheless, the association between enhanced CYP2C19 metabolic activity and the presence of the *CYP2C*:*TG* haplotype, has not reached statistical significance for other CYP2C19 substrates, such as clopidogrel [10] or proton pump inhibitors [16].

Two hypotheses have been proposed to explain the mechanism underlying this haplotype’s effect: (a) CYP2C18 may complement the metabolic capacity of CYP2C19, with this interaction depending on the presence of *CYP2C*:*TG*, due to the high degree of sequence similarity and identity between the two enzymes; or (b) the haplotype may influence *CYP2C19* gene expression at the regulatory level, thereby enhancing enzymatic activity [17].

Furthermore, the *CYP2C*:*TG* has been reported to be associated with ultrarapid CYP2C19 metabolism, similar to that conferred by CYP2C19*17. Therefore, genotyping this haplotype in populations such as Native Americans, where the frequency of CYP2C19*17 is low or nearly absent [13,18], may be useful for predicting gRMs or gUMs phenotypes.

According to a study [8] conducted in a European cohort, the *CYP2C*:*CG* haplotype was the most prevalent, with a frequency of 0.65, compared to 0.16 and 0.19 for the *CYP2C*:*TA* and *CYP2C*:*TG* haplotypes, respectively. These findings are consistent with those reported in European cohorts from the 1000 Genomes Projects [8]. However, they differ from the frequencies observed in the present study, where the *CYP2C*:*TG* haplotype was predominant (42.7%; Table 2). This difference likely reflects the predominantly Native American ancestry (92.12%) of the studied population, in agreement with previous reports showing higher *CYP2C*:*TG* frequencies in four Native American enriched cohorts derived from public databases [12]. The *CYP2C*:*CA* haplotype was not detected in the 1000 Genomes Project database nor in reference European cohorts from previous studies, suggesting that this haplotype is either absent or extremely rare [19]. Its absence was also confirmed in other ethnic groups, including the purely Native American population analyzed here.

The *CYP2C*:*TG* haplotype is found almost exclusively in combination with the CYP2C19*1 allele [20], although it has also been reported in associations with other alleles such as CYP2C19*4 [19]. Our results are consistent with this observation, as the haplotype was found almost exclusively with CYP2C19*1, with the exception of a single individual carrying CYP2C19*2 (Table 1). Moreover, CYP2C19*17 was absent in all *CYP2C*:*TG* homozygotes, which is consistent with previous findings showing the lack of CYP2C19*17+TG diplotype in individuals with gastroesophageal reflux disease treated with omeprazole [19]. Several authors have proposed a linkage disequilibrium between the *CYP2C*:*TG* haplotype and the CYP2C19*1 allele, based on the lack of co-occurrence with either CYP2C19*2 (rs4244285 A) or CYP2C19*17 (rs12248560 T) [8,12]. However, current findings only partially support this assumption, given the single individual identified with the CYP2C19*2+TG combination (Table 1).

When analyzing the impact of the *CYP2C*:*TG* haplotype on the logMR of omeprazole/5-hydroxyomeprazole, a statistically significant difference was observed (*p* = 0.02; Figure 1) between carriers and non-carriers. These results align with a previous study conducted in patients with gastrointestinal disorders treated with omeprazole, where therapeutic failure was associated with the *CYP2C*:*TG*/*TG* diplotype, but not with the increased-function variant CYP2C19*17 [19].

Regarding the analysis of omeprazole logMR across different diplotypes, we observed a trend toward increased CYP2C19-mediated metabolism in carriers of the *CYP2C*:*TG* haplotype, regardless of zygosity, across most metabolizer groups, except for the gRM group (Figure 2). However, this association did not reach statistical significance. These results are consistent with a recently published study that also failed to identify a significant association between *CYP2C*:*TG* or CYP2C19*1+TG and exposure to six drug substrates (pantoprazole, omeprazole, rabeprazole, citalopram, sertraline, and voriconazole) [20].

Conversely, current findings differ from previous reports that identified a significant association of the *CYP2C*:*TG* haplotype with an increased CYP2C19 enzymatic activity in the metabolism of escitalopram and sertraline [8,9]. Those studies suggest that genotyping for the *CYP2C*:*TG* haplotype may enhance therapeutic decision-making and improve phenotype prediction. Indeed, pharmacokinetic data for sertraline indicate that patients homozygous for either CYP2C19*17 or *CYP2C*:*TG*, or carrying both variants, may require higher doses of sertraline compared to CYP2C19*1/*1 carriers to achieve therapeutic plasma concentrations [9].

These discrepancies may be attributed to multiple factors, including the relative contribution of CYP2C19 to the metabolism of escitalopram and sertraline. Other cytochrome P405 enzymes also participate in the metabolism of these drugs [16]. For this reason, the omeprazole/5-hydroxyomeprazole concentration ratio was used in this study, as CYP2C19 is the sole enzyme responsible for this specific metabolic conversion [14,21].

A recent study proposed a phenotype reclassification based on the presence of the *CYP2C*:*TG* haplotype, suggesting that subjects with the CYP2C19*1+TG/*17 genotype should be classified as gUMs rather than gRMs, in accordance with clinical guideline criteria. Similarly, subjects with the CYP2C19*1+TG/*1+TG diplotype would be reclassified as gUMs instead of “normal metabolizers” (gNMs) [12]. However, this genotype-based phenotypic prediction does not align with present findings. Although the presence of the *CYP2C*:*TG* haplotype was observed in gNMs and gIMs individuals it was associated with increased CYP2C19-mediated omeprazole metabolism, but the differences were not statistically significant. Therefore, on the light of present results the presence of *CYP2C*:*TG* alone is not sufficient to reclassify individuals into different metabolizer categories or (predicted phenotypes) (Figure 2).

Furthermore, the genotype–phenotype discrepancy observed in Native American populations has been hypothesized that may be attributable to the presence of the *CYP2C*:*TG* haplotype, although they did not test it by the absence of plasma concentration measurements in that study [15]. Based on present data, the observed genotype–phenotype discordance cannot be explained solely by the presence or absence of the *CYP2C*:*TG* haplotype.

Given that the study was conducted in local health clinics in remote rural areas, there are limitations in controlling the intake of xenobiotics and habits of the participating indigenous Mexican population with deeply rooted traditions. Consequently, a significant limitation of this study is that potential concomitant use of other medications, herbal or traditional medicine remedies, cannot be ruled out, which may have contributed to partial inhibition of CYP450 enzymes [22]. Notably, in the gRM group, the measured enzymatic activity did not align with the phenotype inferred from genotype and the presence of the *CYP2C*:*TG* haplotype, as enzymatic activity was reduced (Figure 2). This discrepancy may be explained by the fact that this group had the highest mean age among the analyzed individuals, which could be associated with age-related decline in hepatic function and a greater likelihood of concomitant use of medication or medicinal plants, factors that are difficult to control in these populations. However, hepatic function tests were within normal parameters, suggesting that another possibility is the presence of an untested or previously undescribed genetic variant. Another limitation of this study is the lack of specific clinical data that could affect CYP2C19 metabolism, such as inflammatory biomarkers (i.e., CRP). Since it has been established that systemic inflammation can reduce CYP2C19 enzymatic activity [23,24]. Considering this, it is difficult to rule out the possibility that reduced CYP2C19 activity observed in some subgroups may be explained by underlying inflammation rather than by the *CYP2C*:*TG* haplotype itself.

This study represents the first analysis of the association between molecular ancestry and the presence or absence of the *CYP2C*:*TG* haplotype. Based on current data, molecular ancestry influences the likelihood of carrying this haplotype cannot be concluded. These findings are consistent with a previous study that reported no significant association between ethnicity and the presence of *CYP2C*:*TG*; however, that study only compared European individuals with a broadly defined “other” category, without specifying ethnic origins [20]. It is important to note that the population analyzed in the present study was predominantly Native American (92%), underscoring the need for future research in more ethnically diverse populations in order to support this observation, that *CYP2C19* genotype frequencies vary across different ethnic groups and consequently metabolic phenotypes [25].

Specifically, when analyzing the presence of various *CYP2C* haplotypes in relation to the molecular ancestry proportions of each individual, we observed a statistically significant difference (*p* = 0.005) between the presence or absence of *CYP2C*:*TA* haplotype and African ancestry (Table 3). However, given the low proportion of African ancestry in our cohort and the limited number of individuals carrying the *CYP2C*:*TA* haplotype, these findings should be interpreted with caution and cannot be generalized to other populations.

The importance of studying the *CYP2C* haplotype as a tool for predicting the CYP2C19 metabolic phenotypes from genotyping data [3] lies in its clinical implications, particularly in contexts where increased CYP2C19 activity has been associated with therapeutic failure or adverse drug reactions, such as in patients undergoing antidepressant treatment [26], those with a history of depressive symptoms [27] or suicide attempts [28]. Furthermore, efforts should be directed toward ensuring the inclusion of non-European populations in pharmacogenomics research, thereby promoting equity in both scientific investigation and clinical practice.

The study of indigenous populations, in this case in the Americas, is essential to prevent widening inequalities and the biotechnology gap. This study is aligned with the need to include ethnicity in clinical research, which is the main motivation behind the RIBEF Network’s study and the basis for the Mérida/T’Hó Declaration issued by this Network together with Council of International Organizations of Medical Sciences (CIOMS) in 2020 [29].

## 4. Materials and Methods

### 4.1. Study Population

Pharmacokinetic data were obtained from a previous analysis [15] involving 256 volunteers (177 women and 79 men) with a mean age ± SD of 39.8 ±15.8 years, recruited from four different rural areas of Mexico (Chihuahua, Durango, Nayarit, and Sonora) [15]. Participants who identified self-identified as Native American were included in this study. Healthy volunteers were included based on routine clinical examination and blood tests. Information on their medical history was collected before the study. Moreover, pregnant women and volunteers with consanguineous relationship were excluded. Volunteers with a history of adverse drug effects or who had taken any medication in the two weeks prior to the study were also excluded. Alcohol, tobacco, and traditional medicine use, as well as concomitant medication, were recorded during the study. The study was approved by the Ethics and Research Committee of the Durango General Hospital, Mexican Health Ministry (CEI-HG450-24/165) and was conducted in accordance with the principles of the Declaration of Helsinki. Written informed consent was obtained from all participants prior to enrollment.

### 4.2. CYP2C19 Alleles and CYP2C Haplotype

Study participants were genotyped for CYP2C19*2, *3, *4, *5, and *17, as previously described [3,15]. Hardy–Weinberg equilibrium was calculated through χ^2^, and *p*-value was determined through 1-pchisq (χ^2^, df = 1). *p* > 0.05 was considered to indicate a Hardy–Weinberg equilibrium. Additionally, genotyping was performed by the same researcher by triplicate for two variants in the *CYP2C18* gene, rs2860840 (C > T) and rs11188059 (G > A), using commercially available TaqMan^®^ gene expression assay (Thermo Fisher Scientific, Waltham, MA, USA) (Table S1). All reactions were performed in 96-well plates using the QuantStudio™ 5 Real-Time PCR System (Applied Biosystems, Waltham, MA, USA) following the manufacturer’s protocol. Genotype-based phenotype predictions for CYP2C19 were assigned as follows: poor metabolizers (gPMs) carry two non-functional alleles, corresponding to an activity score (AS) of 0. Intermediate metabolizers (gIMs) carry either one normal function allele and one non-functional allele, or one increased function allele and one non-functional allele, resulting in AS of 1 to 1.5. Normal metabolizers (gNMs) have two alleles with standard enzymatic activity, yielding an AS of 2. Rapid (gRMs) and ultra-rapid metabolizers (gUMs) exhibit enhanced metabolic capacity due to the presence of one normal function allele paired with an increased-function allele, or two increased-function alleles, respectively [4].

### 4.3. Phenotyping Procedure

A phenotyping protocol (CEIBA cocktail) was developed and validated by the RIBEF network to investigate the indigenous populations of America, which includes probes drugs for CYP2D6, CYP2C9, CYP2C19, CYP3A4, and CYP1A2 [14,30]. In the present study the actual drug-metabolizing capacity was measured using this CEIBA phenotyping approach, which has been previously validated and applied to study populations in Mexico, Ecuador, and Nicaragua [15,31,32]. The participants received single oral doses of omeprazole (20 mg), losartan (25 mg), and caffeine (100 mg) followed one hour later by dextromethorphan (30 mg). Subjects fasted for 12 h before and up to 4 h after the administration of the test drugs. During the study period, participants were instructed to avoid concomitant medications, herbal remedies, over-the-counter drugs, and food products known to induce or inhibit CYP2D6, CYP2C9, CYP2C19, CYP3A4, or CYP1A2. Blood samples were collected 4 h after test drug administration, centrifuged for 10 min at 3500 rpm, and plasma aliquots were stored at −20 °C until analysis by liquid chromatography-tandem mass spectrometry (LC-MS/MS). The MR of omeprazole to 5-hydroxyomeprazole was analyzed to determine individual CYP2C19 functional phenotypes and assess their metabolic capacity [30].

### 4.4. Ancestry

Genomic ancestry for the study participants was previously determined [33]. Briefly, 83 ancestry informative markers (AIMs) were genotyped in all individuals included in this study, as well as in the other populations. Spaniards (*n* = 114) and Native Americans from Peru (*n* = 296) were used as parental European and Native American reference populations, respectively. The Yoruba individuals (*n* = 119) from the 1000 Genomes Project [34] were used as the African reference population. Admixture software (v. 1.3.0) [35] was used to perform individual ancestry analyses in an unsupervised mode, assuming a tri-hybrid model (k = 3).

### 4.5. Statistical Analysis

#### 4.5.1. Analysis of the Impact of the *CYP2C:TG* Haplotype on the logMR Levels

To explore the potential impact of the *CYP2C*:*TG* haplotype on logMR levels, a univariate statistical analysis was performed. Initially, logMR values were visualized across individuals, or stratified based on the presence or absence of the TG haplotype. Subsequently, the Wilcoxon rank-sum test was applied to assess whether statistically significant differences existed between the two groups. This non-parametric test was selected due to the non-normal distribution of the variable under study. Statistical significance was defined as *p* < 0.05. Additionally, boxplots were generated to illustrate the variation in logMR levels as a function of the combined *CYP2C19* genotypes, their predicted metabolic phenotypes, and the presence or absence of the *CYP2C*:*TG* haplotype. All univariate analyses were performed in R (version 4.4.3), using the *ggplot2*, *ggsignif*, and *stats* packages. Moreover, to obtain an integrated view of the combined effect of the variables considered, we performed a multivariate analysis using a generalized linear model the relative contribution of each predictor using the LMG metric implemented in the *relaimpo* R package (version 2.2.7) [36].

#### 4.5.2. Analysis of the Relationship Between Ancestry and *CYP2C* Haplotypes

To assess the relationship between genomic ancestry levels and the presence of various *CYP2C* haplotypes, a univariate statistical analysis was conducted. The ancestry components considered included NAT (proportion of Native American ancestry), EUR (European ancestry) and AFR (African ancestry). For each ancestry component, comparisons were made between individuals carrying versus not carrying the respective *CYP2C* haplotypes. The Wilcoxon rank-sum test was applied to determine whether the distribution of each proportion significantly differed between carriers of one of the *CYP2C* haplotypes and those who did not.

All analyses were performed using R, using the *ggplot2*, *ggsignif*, and *stats* packages.

#### 4.5.3. Inference of Individual Haplotypes and Diplotypes

Individual haplotypes and diplotypes were inferred using the HaploStats software (version 1.9.7) package within the R environment [37]. This tool assigns posterior probability values to each individual’s diplotype configuration based on estimated haplotype frequencies. For inclusion in the analysis, a minimum posterior probability threshold of 0.90 was applied for inclusion in the analysis.

## 5. Conclusions

The present study expands the limited evidence regarding the role of the *CYP2C*:*TG* haplotype in CYP2C19-mediated drug metabolism, particularly among non-European populations such as Mexican Indigenous cohort analyzed here. Although these findings do not yet support the clinical implementation of *CYP2C*:*TG* genotyping, they suggest that the mechanism by which the *CYP2C*:*TG* haplotype may enhance CYP2C19-dependent metabolism warrants further investigation across ethnically diverse populations, especially considering the variability in *CYP2C19* genotype frequencies among different ethnic groups and its implications for metabolic phenotype prediction.

## Figures and Tables

**Table 1:** Distribution of *CYP2C19* alleles frequencies according to *CYP2C* haplotypes (*CYP2C*:*CG*, *CYP2C*:*TG*, *CYP2C*:*TA*). Values are expressed as the number of alleles and their relative percentage (%).

*CYP2C* Haplotypes	*CYP2C19* Alleles (*n*, %)		
	*1	*17	*2
***CG***	113 (22.07)	9 (1.76)	61 (11.91)
***TG***	218 (42.58)	0 (0.00)	1 (0.19)
***TA***	110 (21.48)	0 (0.00)	0 (0.00)

**Table 2:** Demographic, genetic, and phenotypic characteristics across *CYP2C* diplotype subgroups.

*CYP2C* Diplotypes	Phenotypes	Subjetcs, n, %	Sex		Age, Years, Mean (sd)	LogMR, Mean (sd)
			Men %	Women %		
***TG*** **haplotypes**						
*1+TG/*17	gRM	4 (1.6)	50	50	55 (26.9)	0.79 (0.47)
*1+TG/*1+TG	gNM	46 (17.9)	30.4	69.6	41.5 (17.7)	0.25 (0.40)
*1/*1+TG	gNM	96 (37.7)	20.2	69.8	38.7 (15.2)	0.26 (0.40)
*1+TG/*2+TG	gIM	1 (0.4)	100	0		
*1+TG/*2	gIM	25 (9.7)	40	60	34.6 (14.9)	0.46 (0.30)
***CG* or *TA* haplotypes**						
*1/*17	gRM	5 (1.9)	80	20	42.8 (1.48)	−0.03 (0.35)
*1/*1	gNM	49 (19.1)	18.4	81.6	38.6 (14.9)	0.3 (0.38)
*1/*2	gIM	24 (9.3)	38	62	43.9 (16.0)	0.62 (0.39)
*2/*2	gPM	6 (2.3)	16.7	83.3	45.5 (15.1)	1.25 (0.34)

**Table 3:** Comparison of ancestry proportions (AFR, NAT, EUR) according to the presence or absence of *CYP2C* haplotypes (*CYP2C*:*TG*, *CYP2C*:*TA*, *CYP2C*:*CG*).

	*CYP2C* Haplotypes								
	*TG*			*TA*			*CG*		
Ancestry	Mean (sd)		*p*-Value	Mean (sd)		*p*-Value	Mean (sd)		*p*-Value
	No	Yes		No	Yes		No	Yes	
AFR	0.87 (1.67)	1.22 (2.15)	0.35	1.33 (2.16)	0.74 (1.67)	0.005	0.92 (1.86)	1.24 (2.10)	0.13
NAT	92.5 (8.67)	91.9 (10.1)	0.80	91.7 (9.76)	92.8 (9.53)	0.06	92.3 (9.88)	92.0 (9.55)	0.32
EUR	6.62 (8.52)	6.85 (9.46)	0.98	6.98 (9.33)	6.44 (8.89)	0.36	6.78 (9.00)	6.77 (9.28)	0.81

## Data Availability

The raw data supporting the conclusions of this article will be made available by the authors on request.

## Supplementary Materials

The following supporting information can be downloaded at: https://www.mdpi.com/article/10.3390/ph19010006/s1, Table S1. *CYP2C19* variants and their corresponding TaqMan gene expression assays utilized for Real-Time PCR genotyping.

## Abbreviations

The following abbreviations are used in this manuscript:

AS	Activity score
gIMs	Intermediate metabolizers
gNMs	Normal metabolizers
gPMs	Poor metabolizers
gRMs	Rapid metabolizers
gUMs	Ultrarapid metabolizers
logMR	Metabolic ratio logarithm
MR	Metabolic ratio

## Appendix A

### RIBEF-IBEROFEN Consortium for the Study of Phenotype-Genotype Relationships and Ancestry in Native American Populations

Adrián Llerena ^1,2,3,4^, Carla González de la Cruz ^1,2,4^, Carmen Mata-Martín ^1,2,4^, Juan Antonio Villatoro-García ^1,2,4^, Eva Peñas-Lledó ^1,2,3^, Pedro Dorado ^1,2,3^, Fernando de Andrés ^1,5^, Nadine de Godoy Torso ^1,2,6^, Fernanda Rodrigues-Soares ^1,2,7^, Martha Sosa-Macías ^1,8^, Carlos Galaviz-Hernández ^1,8^, Enrique Terán ^1,9^, Catalina Altamirano-Tinoco ^1,10^, Ronald Ramírez-Roa ^1,11^.

1. RIBEF/SIFF Red y Sociedad Iberoamericana de Farmacogenética y Farmacogenómica. Spain
2. INUBE University Institute for Bio-Sanitary Research of Extremadura. Badajoz, Spain.
3. Faculty of Medicine and Health Sciences, University of Extremadura. Badajoz, Spain.
4. Unit of Pharmacogenomics and Personalized Medicine. Clinical Pharmacology Service. Badajoz University Hospital, SES. Badajoz, Spain.
5. Current address: Department of Analytical Chemistry and Food Technology. Faculty of Pharmacy, University of Castilla-La Mancha. Albacete, Spain.
6. Facultad de Ciencias Médicas, Universidade de Campinas. Campinas, Sao Paulo, Brasil.
7. Department of Pathology, Genetic and Evolution, Universidade Federal do Triângulo Mineiro, Uberaba, Brasil.
8. IPN Instituto Politécnico Nacional. CIIDIR Unidad Durango. Academia de Genómica. Durango, México.
9. Colegio de Ciencias de la Salud, Universidad San Francisco de Quito, Quito, Ecuador.
10. UNAN Universidad Nacional Autónoma de Nicaragua, Facultad de Ciencias Médicas, León, Nicaragua.
11. Facultad de Odontología, Universidad Americana, Managua, Nicaragua.
