# Supplementary material for: CYP2C:TG Haplotype in Native Mexicans, Molecular Ancestry and Its Implications for CYP2C19 Genotype–Phenotype Correlation
Source: Pharmaceuticals (Basel). 2025 Dec 19;19(1):6. doi: 10.3390/ph19010006 (PMC12844601; doi:10.3390/ph19010006)
Supplement: Supplementary file 1 [file pharmaceuticals-19-00006-s001.zip › pharmaceuticals-4003704-supplementary.pdf]

Table S1. *CYP2C19* variants and their corresponding TaqMan gene expression assays utilized for Real-Time PCR genotyping.

CYP gene	Allelic variant	rs ID	Nucleotide change	Allele Functional Status	TaqMan Assay ID
CYP2C19	*2	rs4244285	19154G>A	None	C__25986767_70
		rs12769205	12662A>G		C__25744790_10
	*3	rs4986893	17948G>A	None	C__27861809_10
	*4	rs28399504	1A>G	None	C__30634136_10
	*5	rs56337013	90033C>T	None	C__27861810_10
	*17	rs12248560	-806C>T	Increased	C____469857_10
CYP2C haplotype		rs2860840	31C>T		C__11201742_10
		rs11188059	819+2182G>A		C__31983321_10
